# Tubulointerstitial nephritis uveitis syndrome with Fanconi syndrome: Two case reports and literature review

**DOI:** 10.1097/MD.0000000000046202

**Published:** 2025-11-21

**Authors:** Jingjing Lin, Jia Sun

**Affiliations:** a Department of Nephrology, Linping Campus, The Second Affiliated Hospital of Zhejiang University School of Medicine, Hangzhou, People’s Republic of China.

**Keywords:** case report, Fanconi syndrome, tubulointerstitial nephritis, uveitis

## Abstract

**Rationale:**

Tubulointerstitial nephritis uveitis syndrome (TINU) is a rare form of acute interstitial nephritis accompanied by uveitis. However, few cases of TINU associated with Fanconi syndrome (FS) have been reported, and the clinical characteristics and optimal management of this rare association remain poorly defined.

**Patient concerns:**

In this case report, we aimed to describe 2 cases of TINU associated with FS and their management, emphasizing the importance of recognizing FS as a potential complication in patients with TINU.

**Diagnoses:**

Two adult patients with TINU and FS were evaluated. Both presented with renal insufficiency, hypouricemia, hypophosphatemia, hypokalemia, renal glycosuria, low-molecular-weight proteinuria, and uveitis. One patient also had renal tubular acidosis. Renal biopsy revealed acute interstitial nephritis in both cases, with one case additionally showing immunoglobulin A nephropathy.

**Interventions/outcomes:**

Systemic and ocular topical corticosteroids were administered, and potassium citrate was used to correct renal tubular acidosis. Renal function, electrolyte levels, and urinalysis results normalized in both patients.

**Lessons:**

In patients with TINU and renal tubular dysfunction (such as low-molecular-weight proteinuria, hypophosphatemia, and hypouricemia) the possibility of secondary FS should be considered. Along with corticosteroid therapy, timely treatment of renal tubular acidosis is essential.

## 1. Introduction

Tubulointerstitial nephritis uveitis syndrome (TINU) is characterized by acute tubulointerstitial nephritis (ATIN) accompanied by uveitis. Clinically, TINU often presents with nonspecific systemic symptoms, including fever, anorexia, weight loss, fatigue, abdominal or lumbar pain, arthralgia, and headache. Uveitis is usually bilateral (80%)^[1]^ and predominantly anterior. Renal involvement commonly manifests as proteinuria and renal insufficiency and may include renal tubular dysfunction such as hypokalemia and hypophosphatemia. Fanconi syndrome (FS) is a generalized proximal tubular dysfunction characterized by renal glycosuria, aminoaciduria, phosphaturia, uricosuria, low-molecular-weight proteinuria, and type II renal tubular acidosis. FS is most often observed in adults and can occur secondary to certain medications (e.g., aminoglycosides and cyclophosphamide), heavy metal poisoning, or abnormal secretion and reabsorption of monoclonal immunoglobulin (Ig) light chains in the proximal tubules. FS is less commonly observed in patients with autoimmune interstitial diseases such as Sjögren’s syndrome and primary biliary cholangitis. To date, only a few cases of TINU with FS have been reported. Here, we describe the diagnosis and management of 2 such cases.

## 2. Case presentation

### 2.1. Case 1

A 51-year-old man was admitted to the hospital on May 27, 2021, with fatigue for 1 month. He also reported photophobia and ophthalmalgia accompanied by a 4 kg weight loss. On admission, physical examination revealed bilateral conjunctival hyperemia and a blood pressure of 147/86 mm Hg. Laboratory tests showed renal insufficiency, hyperglobulinemia, and mild anemia (Table 1). Tests for autoimmune disease markers (including Ig light chains in blood and urine, antinuclear antibodies, and antineutrophil cytoplasmic antibodies) as well as immunofixation electrophoresis and tests for infectious diseases (e.g., hepatitis and syphilis) were all negative. Ophthalmological examination confirmed anterior uveitis.

**Table 1:** Comparison of patients’ laboratory test results before and after treatment.

Examination item	Case 1		Case 2		Reference value
	Before	After	Before	After	
Serum biochemistry					
Potassium (mmol/L)	3.42	4.05	2.84	3.83	3.50–5.50
Phosphorus (mmol/L)	0.78	1.05	0.62	1.18	0.81–1.45
Calcium (mmol/L)	2.31	2.39	2.32	2.42	2.08–2.60
Sodium (mmol/L)	140.0	141.1	139.4	139.1	135.0–145.0
Chloride (mmol/L)	105.4	103.4	105.0	106.4	96.0–106.0
Blood glucose (mmol/L)	5.62	6.0	5.23	4.63	3.89–6.11
Creatinine (μmol/L)	159.0	94	165.0	85	40.0–88.0
Albumin (g/L)	36.9	45.9	41.7	41.2	35.0–52.0
Globulin (g/L)	39.0	25.9	36.8	30.3	15.0–30.0
Uric acid (μmol/L)	139.0	278	105.0	253	154.0–357.0
Erythrocyte sedimentation (mm/h)	71	8	42	–	0–20
Hemoglobin (g/L)	114	163	86	125	113–151
IgG4 (g/L)	0.78	–	2.94	–	0.03–2.01
Arterial blood gas analysis					
pH value	7.40	–	7.354	7.364	7.35–7.45
Base excess (mmol/L)	−0.6	–	−8	–	−2.0 to 3.0
HCO_3_^−^ (mmol/L)	24.2	–	16.6	23.7	22.0–27.0
PaCO_2_ (mm Hg)	39	–	3.6	42.5	35.0–48.0
Urinalysis					
pH	6.5	6.0	7.0	6.0	5.4–8.4
Proteinuria (g/L)	1+	negative	2+	negative	negative
Urine glucose	1+	negative	2+	negative	negative
Total protein to creatinine ratio (mg/g.Cr)	808.3	39.2	–	–	0.00–150.00
24 hours proteinuria (mg/24 h)	845.1	–	1036	–	28.0–141
24 hours potassium (mmol/24 h)	49.9	–	48.83	–	25–100
24 hours uric acid (μmol/24 h)	–	–	1325	–	1488–4463
Spot urinary potassium/creatinine (mmol/mmol)	–	–	–	–	–
24 hours phosphorus (mmol/24 h)	–	–	12.47	–	12.90–42.00
24 hours sodium (mmol/24 h)	–	–	184.76	–	130–260
24 hours calcium (mmol/24 h)	–	–	2.53	–	0.25–6.2
Uric acid excretion fraction	–	–	43.55	–	2–10%
Uric phosphorus excretion fraction	–	–	25.74	–	5–15%

“–” indicates value not measured or undetected.

HCO_3_^−^ = bicarbonate, IgG4 = immunoglobulin, PaCO_2_ = partial pressure of carbon dioxide.

**Figure 1.**
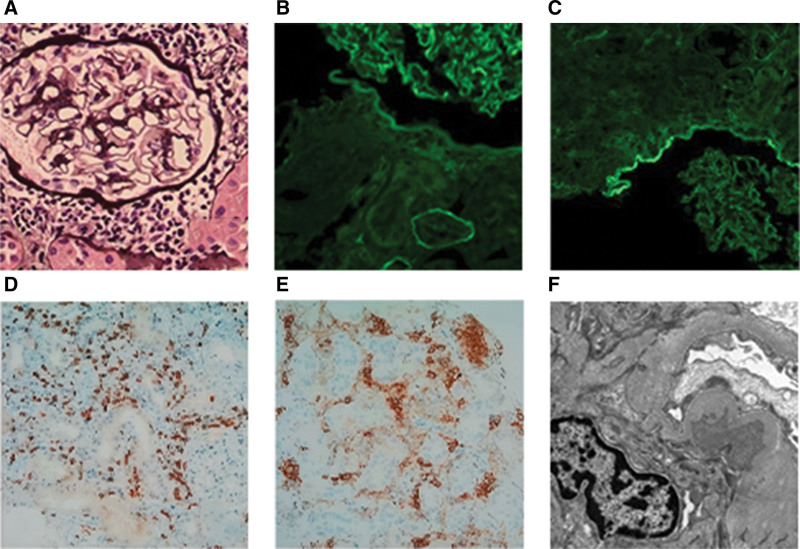
Renal pathology of Case 1. (A) Light microscopy shows partial dilation of renal tubule lumens, epithelial cell detachment, significant interstitial edema, and infiltration by numerous lymphocytes, monocytes, and plasma cells (PASM, ×400). (B and C) Mesangial deposits of IgA (++) (B) and IgM (+) (C) (immunofluorescence, ×200). (D and E) Immunohistochemistry shows CD3+: multifocal lymphocyte positivity (D) and CD20+: focal lymphocyte positivity (E). (F) Electron microscopy reveals partial foot process effacement and electron-dense deposits in the mesangial area (×5000). Ig = immunoglobulin, PASM = periodic acid-schiff-methenamine.

Renal biopsy revealed findings consistent with ATIN and IgA nephropathy (Fig. 1). The patient also exhibited hypouricemia, hypokalemia, hypophosphatemia, low-molecular-weight proteinuria, and renal glycosuria, consistent with FS. Based on the pathological and ophthalmological findings, a diagnosis of TINU with FS was made.

Treatment involved oral prednisolone at an initial dose of 50 mg once daily, tapered systematically over approximately 9 months. During treatment, the patient experienced recurrent uveitis, which responded to topical corticosteroids. At the final follow-up, 11 months after discharge (2 months after completing a 9-month course of corticosteroid therapy), the patient’s renal function had significantly improved, with serum creatinine stabilizing at 94 μmol/L and estimated glomerular filtration rate recovering to 80 mL/min/1.73 m^2^. Electrolyte disturbances had resolved, with serum potassium, phosphorus, and uric acid levels maintained within normal ranges without supplementation. Repeat urinalysis showed no protein, glucose, or abnormal cellular elements.

### 2.2. Case 2

A 52-year-old woman was admitted to the hospital on December 6, 2017, with poor appetite, xerostomia, and increased nocturia for 2 months, along with ocular itching, redness, blurred vision, and a 14-kg weight loss. On admission, physical examination revealed conjunctival hyperemia in the right eye. Laboratory tests demonstrated renal insufficiency, hypergammaglobulinemia, and anemia. Tests for antinuclear antibodies, antineutrophil cytoplasmic antibodies, and hepatitis markers, along with immunofixation electrophoresis, showed no significant abnormalities. Ophthalmological examination revealed bilateral anterior uveitis and dry eye syndrome.

**Figure 2.**
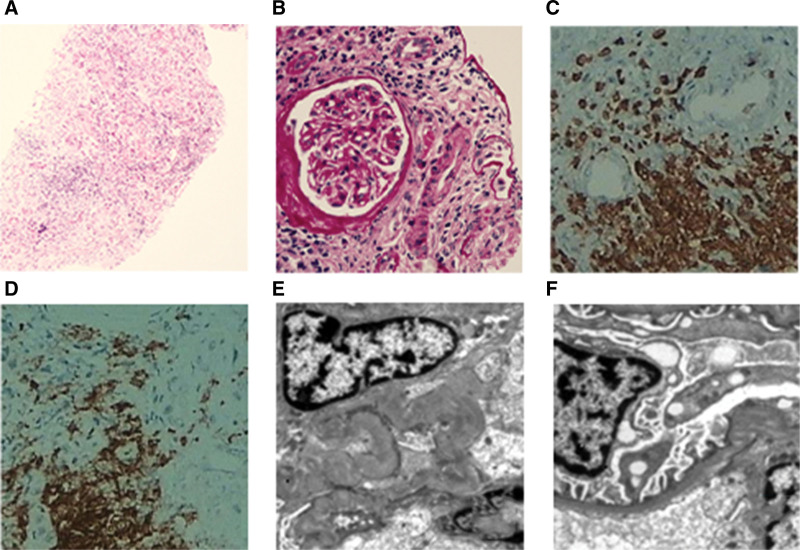
Renal pathology of Case 2. (A and B) Light microscopy shows thickening of some renal tubular basement membranes, reduced luminal size, interstitial edema, and multifocal inflammatory cell infiltration (A: hematoxylin and eosin, ×100; B: periodic acid-Schiff, ×400). Immunohistochemistry shows CD3+: multifocal lymphocyte positivity (C) and CD20+: focal lymphocyte positivity (D). (E and F) Electron microscopy reveals extensive foot process effacement and interstitial inflammatory cell infiltration (×5000).

Renal biopsy showed ATIN (Fig. 2). The presence of hypouricemia, hypophosphatemia, renal glycosuria, low-molecular-weight proteinuria, and proximal tubular dysfunction with markedly increased urinary phosphorus and uric acid excretion confirmed FS. The patient also had hypokalemic hyperchloremic metabolic acidosis, suggesting concurrent type II renal tubular acidosis. Based on the renal pathology and ophthalmological findings, a diagnosis of TINU with FS was established.

The patient was treated with oral potassium citrate (10%) for potassium supplementation, along with topical tobramycin, dexamethasone, pranoprofen, and compound tropicamide eye drops. Beginning December 11, 2017, intravenous methylprednisolone (40 mg once daily) was administered, later switched to oral methylprednisolone with gradual tapering until discontinuation. At the final follow-up, 17 months after discharge (7 months after completing a 10-month course of corticosteroid therapy), the patient’s renal function had recovered, with serum creatinine stabilizing at 85 μmol/L and estimated glomerular filtration rate at 68 mL/min/1.73 m^2^. The metabolic acidosis had resolved, and potassium citrate supplementation was discontinued. Repeat urinalysis showed no protein, glucose, or abnormal cellular elements.

## 3. Discussion and conclusion

In this study, 2 middle-aged patients presented with acute kidney injury and uveitis. Renal biopsy demonstrated ATIN, and laboratory tests revealed abnormalities such as hypokalemia, hypophosphatemia, hypouricemia, renal glycosuria, and low-molecular-weight proteinuria, consistent with FS. Thus, a diagnosis of TINU with FS was established. Following treatment with systemic and ocular topical corticosteroids and correction of acidosis, the patients’ renal function, electrolyte levels, and urinalysis normalized.

TINU, first described in 1975,^[2]^ is rare. In specialized uveitis centers, its incidence ranges from 0.1% to 2.0%. Over the past 40 years, more than 300 cases have been reported worldwide, with approximately 60% occurring in children. The differential diagnosis of TINU should exclude infectious and autoimmune diseases. The diagnosis is often one of exclusion, requiring consideration of other causes of acute interstitial nephritis. Notably, crystalline nephropathy, such as oxalate crystal deposition, represents an important and often overlooked category of acute interstitial nephritis, as highlighted in a recent case report by Lathiya et al.^[3]^ In a Chinese adult cohort, TINU accounted for 28% of ATIN cases (significantly higher than the 4.7% reported in the literature).^[4]^ The median age of onset of TINU is 15 years (range, 9–74 years),^[1]^ and the average age of adult patients is 45 years. The reported male-to-female ratios are 1:2.5 and 1:5.^[4]^

The etiology of TINU remains unclear. Research suggests that cellular and humoral immunity play a major role in its pathogenesis, with external infections triggering an autoimmune response affecting the eyes and kidneys.^[5]^ Uveal and renal tubular epithelial cells share a common monomeric C-reactive protein autoantigen, and patients with TINU have been found to have high titers of anti-monomeric C-reactive protein antibodies, which may trigger an immune response.^[6]^

TINU is characterized by immune-mediated inflammatory cell infiltration of the renal interstitium.^[7]^ Uveitis can occur up to 2 months before or 14 months after kidney injury. Patients with renal involvement often present with mild-to-moderate low-molecular-weight proteinuria (usually <2 g/d), and urine sediment microscopy may reveal red blood cells, white blood cells, or granular casts. Uveitis is commonly accompanied by moderate-to-severe non-oliguric acute renal failure with tubular dysfunction.

However, cases of TINU accompanied by FS are rare. To date, 11 publications have reported 12 cases,^[8–18]^ summarized as follows: among the 12 patients, there were 9 adults (8 women) and 3 children (2 boys), with a median age of onset of 43 years (range, 9–57 years). The average age of the adult patients was 45 years, with 6 (50%) being middle-aged women. Four cases were reported in Japan,^[10,11,14,15]^ with others from the United States, France, South Korea, Morocco, and additional countries. The initial presentation was tubulointerstitial nephritis or uveitis, often accompanied by systemic symptoms such as fatigue, malaise, nausea, and weight loss. Renal manifestations typically included moderate-to-severe renal insufficiency and proximal tubular defects. Most cases also presented with mild hypokalemia, hypophosphatemia, and hypouricemia, with metabolic acidosis reported in 5 cases and occasional secondary distal tubular dysfunction. Leukocyturia was common, and hematuria was also observed. Three patients had elevated antistreptolysin O titers,^[12,13,15]^ with 2 reporting prior antibiotic use.^[12,15]^ One patient had herpes zoster^[16]^ and a history of nonsteroidal anti-inflammatory drug use. In a 13-year-old girl from Japan,^[11]^ anti-tubular basement membrane antibodies were detected, whereas a 46-year-old woman from South Korea was HLA-B27 positive, although there was no evidence of ankylosing spondylitis.^[18]^ Eight patients received systemic corticosteroid treatment,^[8,9,11,15–18]^ which improved renal function, although mild tubular dysfunction persisted in some cases. Uveitis recurred in 4 cases.^[9,13,15,18]^

Unlike the characteristic predominance of TINU in children and adolescents, TINU with FS occurs more frequently in middle-aged women. Currently, there is no evidence of a racial or ethnic predisposition.

Tubular damage in acute interstitial nephritis is a focal lesion in which infiltrating inflammatory cells penetrate and disrupt the tubular basement membrane, causing injury to adjacent epithelial cells that contain numerous energy-producing mitochondria. Cellular ATP depletion can inhibit Na^+^/K^+^-ATPase and impair reabsorption processes, suggesting that mitochondrial dysfunction may be a key mechanism in FS pathogenesis.^[19]^ Furthermore, infiltrating lymphocytes can directly contact tubular epithelial cells, often leading to endothelial damage and explaining the tubular dysfunction observed in many cases.^[20]^

TINU is rare, and no standardized treatment protocol has been established. Most reported cases have been treated with corticosteroids or other immunomodulators.^[5]^ Corticosteroids can be administered orally or intravenously, typically at doses of 1 to 1.5 mg/kg/d, with tapering regimens varying according to the degree of remission and clinician experience. Uveitis generally responds well to local or systemic corticosteroid treatment; however, recurrence is common. In this study, recurrence occurred in the first patient and resolved with local corticosteroid therapy. In a 2014 cohort study^[4]^ of 31 adult Chinese patients with TINU, despite active immunotherapy, 92% had residual renal insufficiency and/or tubular dysfunction after 12 months of treatment. Older age, leukocyturia, elevated serum creatinine, high erythrocyte sedimentation rate, and concurrent thyroid disease were associated with poor renal prognosis. Reports of TINU with FS remain rare, and the relatively short follow-up time makes prognosis difficult to assess. Nonetheless, both patients in this study achieved complete remission with favorable outcomes.

TINU with FS is rare and appears more common in middle-aged women. Nevertheless, the findings of this study should be interpreted in the context of its limitations. First, as a case report of only 2 patients, the generalizability of our findings is inherently limited. Second, the follow-up period, although demonstrating full recovery, was of intermediate duration; therefore, the long-term outcomes and potential for late recurrence remain unknown. Finally, we were unable to perform advanced immunologic studies to further elucidate the precise pathogenic mechanism linking TINU and FS. Despite these limitations, our report contributes to the understanding of this rare condition. In diagnosing TINU, the possibility of secondary FS should be considered if there is evidence of tubular dysfunction such as low-molecular-weight proteinuria, hypophosphatemia, or hypouricemia. For treatment, in addition to systemic corticosteroids, renal tubular acidosis should be corrected with potassium citrate.

## Author contributions

**Conceptualization:** Jingjing Lin, Jia Sun.

**Data curation:** Jingjing Lin.

**Formal analysis:** Jingjing Lin.

**Investigation:** Jingjing Lin, Jia Sun.

**Methodology:** Jingjing Lin.

**Project administration:** Jingjing Lin, Jia Sun.

**Resources:** Jia Sun.

**Software:** Jingjing Lin.

**Supervision:** Jingjing Lin, Jia Sun.

**Validation:** Jingjing Lin.

**Visualization:** Jingjing Lin.

**Writing – original draft:** Jingjing Lin.

**Writing – review & editing:** Jingjing Lin, Jia Sun.

## References

[1] Mandeville JT Levinson RD Holland GN. The tubulointerstitial nephritis and uveitis syndrome. Surv Ophthalmol. 2001;46:195–208. 11738428 10.1016/s0039-6257(01)00261-2

[2] Dobrin RS Vernier RL Fish AL. Acute eosinophilic interstitial nephritis and renal failure with bone marrow-lymph node granulomas and anterior uveitis. A new syndrome. Am J Med. 1975;59:325–33. 1163543 10.1016/0002-9343(75)90390-3

[3] Lathiya MK Errabelli P Roy S Mareedu N. Severe acute kidney injury due to oxalate crystal induced severe interstitial nephritis: a case report. World J Nephrol. 2024;13:93976. 38983760 10.5527/wjn.v13.i2.93976 PMC11229832

[4] Li C Su T Chu R Li X Yang L. Tubulointerstitial nephritis with uveitis in Chinese adults. Clin J Am Soc Nephrol. 2014;9:21–8. 24202135 10.2215/CJN.02540313 PMC3878694

[5] Clive DM Vanguri VK. The syndrome of tubulointerstitial nephritis with uveitis (TINU). Am J Kidney Dis. 2018;72:118–28. 29429748 10.1053/j.ajkd.2017.11.013

[6] Tan Y Yu F Qu Z. Modified C-reactive protein might be a target autoantigen of TINU syndrome. Clin J Am Soc Nephrol. 2011;6:93–100. 20813859 10.2215/CJN.09051209 PMC3022254

[7] Amaro D Carreño E Steeples LR Oliveira-Ramos F Marques-Neves C Leal I. Tubulointerstitial nephritis and uveitis (TINU) syndrome: a review. Br J Ophthalmol. 2020;104:742–7. 31719109 10.1136/bjophthalmol-2019-314926

[8] Legendre M Devilliers H Perard L. Clinicopathologic characteristics, treatment, and outcomes of tubulointerstitial nephritis and uveitis syndrome in adults: a national retrospective strobe-compliant study. Medicine (Baltimore). 2016;95:e3964. 27367994 10.1097/MD.0000000000003964 PMC4937908

[9] Vô B Yombi JC Aydin S Demoulin N Yildiz H. TINU-associated Fanconi syndrome: a case report and review of literature. BMC Nephrol. 2018;19:274. 30340545 10.1186/s12882-018-1077-0 PMC6194638

[10] Igarashi T Kawato H Kamoshita S Nosaka K Seiya K Hayakawa H. Acute tubulointerstitial nephritis with uveitis syndrome presenting as multiple tubular dysfunction including Fanconi’s syndrome. Pediatr Nephrol. 1992;6:547–9. 1482643 10.1007/BF00866499

[11] Wakaki H Sakamoto H Awazu M. Tubulointerstitial nephritis and uveitis syndrome with autoantibody directed to renal tubular cells. Pediatrics. 2001;107:1443–6. 11389273 10.1542/peds.107.6.1443

[12] Lessard M Smith JD. Fanconi syndrome with uveitis in an adult woman. Am J Kidney Dis. 1989;13:158–9. 2916570 10.1016/s0272-6386(89)80136-2

[13] Llorente-Gómez B González-Menchén C de Lucas-Collantes C. Tubulo-interstitial nephritis and uveitis with Fanconi syndrome. Nefrologia. 2012;32:263–4. 22466281 10.3265/Nefrologia.pre2011.Dec.11238

[14] Takeda M Hirata M Koni I Miyazaki R Tohuku Y Takeda R. A case of renal-ocular syndrome with Fanconi syndrome [in Japanese]. Nihon Jinzo Gakkai Shi. 1988;30:103–9. 3386007

[15] Koike K Lida S Usui M. Adult-onset acute tubulointerstitial nephritis and uveitis with Fanconi syndrome. case report and review of the literature. Clin Nephrol. 2007;67:255–9. 17474563 10.5414/cnp67255

[16] Yao YH Lin CC Chung YM. Tubulointerstitial nephritis and uveitis syndrome (TINU) with Fanconi’s syndrome. Clin Nephrol. 2011;75(Suppl 1):75–8. 21269599 10.5414/cn106471

[17] Wen YK. Tubulointerstitial nephritis and uveitis with Fanconi syndrome in a patient with ankylosing spondylitis. Clin Nephrol. 2009;72:315–8. 19825339 10.5414/cnp72315

[18] Kim M Kim HW Kim JY. A case of tubulointerstitial nephritis and uveitis with Fanconi syndrome [in Korean]. Korean J Med. 2015;88:711–4.

[19] Heidari R. The footprints of mitochondrial impairment and cellular energy crisis in the pathogenesis of xenobiotics-induced nephrotoxicity, serum electrolytes imbalance, and Fanconi’s syndrome: a comprehensive review. Toxicology. 2019;423:1–31. 31095988 10.1016/j.tox.2019.05.002

[20] Raghavan R Eknoyan G. Acute interstitial nephritis – a reappraisal and update. Clin Nephrol. 2014;82:149–62. 25079860 10.5414/CN108386 PMC4928030

